# Investigation on the Micro Deformation Mechanism of Asphalt Mixtures under High Temperatures Based on a Self-Developed Laboratory Test

**DOI:** 10.3390/ma13071791

**Published:** 2020-04-10

**Authors:** Jilu Li, Wei Guo, Anxin Meng, Meizhao Han, Yiqiu Tan

**Affiliations:** 1 School of Transportation Science and Engineering, Harbin Institute of Technology, Harbin 150090, China; lijiluhit@163.com (J.L.); menganxinhit@163.com (A.M.); hmzsourire@163.com (M.H.); 2 School of Transportation, Jilin University, Changchun 130022, China; guowei17@mails.jlu.edu.cn

**Keywords:** rutting, mechanism, reduced scale circular track test, discrete element, gradation

## Abstract

Rutting has always been considered the main disease in asphalt pavement. Dealing with rutting disease would be benefitted by understanding the formation of rutting and testing the rutting performance of mixtures more reasonably. The objective of this paper is to systematically investigate the rutting mechanism by employing a self-designed rutting tester along with the corresponding numerical simulations. The deformation of different positions of the existing tracking tester was found to be inconsistent, and the loading was not in line with reality. Accordingly, a more practical tester was proposed: the reduced scale circular tracking (RSCT) tester integrates the functions of asphalt mixture fabrication and rutting monitoring. The results demonstrated that the loading of the new tester is closer to the actual situation. In addition, determining the stress and displacement characteristics of particles in the asphalt mixture was found to be difficult due to the limitations of the testing methods. Therefore, a two-dimensional virtual rutting test based on the RSCT was built using PFC2D (Particle Flow Code 2 Dimension) to investigate the mechanism of formation in rutting and to obtain the corresponding guidance. The numerical simulation showed that all particles of the specimen tended to move away from the load location. The main cause of rutting formation was the eddy current flow of asphalt mastic driven by coarse aggregates. The aggregates with diameters ranging from 9.5 to 4.75 mm were observed to have the greatest contribution to rutting deformation. Therefore, the aggregate amount of these spans should be focused on in the design of mixture grading.

## 1. Introduction

Asphalt pavement possesses superior qualities in performance, such as its surface smoothness, low noise, convenient construction and maintenance, and has been widely used around the world [1,2,3,4,5]. With the aggravation of traffic loads and the continuous rise in global average temperatures, rutting disease of asphalt pavement under high temperatures has been a focus for road researchers [6,7,8,9]. The existence of ruts damages the flatness of the road surface, easily causing a vehicle to slip and detrimentally affecting the comfort of drivers. Accurate rutting tests and reasonable evaluation methods serve as the basis to solve the rutting problem.

Currently, the full-scale pavement test and the laboratory rutting test are two major methods that characterize the rutting performance of asphalt mixtures. Full-scale pavement tests include the NCAT (National Center for Asphalt Technology test road) [10], the AASHO (American Association of State Highway Officials**)** test road [11], Minnesota Road, Wes Track [12], the MLS (Mobile Loading Simulator) [13], the HVS (Heavy Vehicle Simulator) [14], the ALF (Accelerated Loading Facility) [15], and so forth. These rutting tests accurately evaluate the rutting performance of asphalt pavement using the actual load, which conforms to the actual situation well. However, both the human and material resources consumed are too large, and it is difficult to repeat the tests many times. The laboratory rutting tests include the HWTT (Hamburg Wheel Tracking Test) [16,17] and the WTT (Wheel Tracking Test) [18,19]. Linear loading is the main loading form in laboratory tests. Due to the limitations in the working principle of the tester, the linear loading mode will cause different deformations of asphalt mixture at different positions. Moreover, the loading speeds of the HWTT and WTT are 0.9984 and 0.5796 km/h, respectively, far from the actual vehicular speed. Accordingly, the aforementioned empirical tests insufficiently characterize the rutting resistance behaviors of asphalt mixtures. Thus, developing a new tester is necessary in order to investigate the rutting mechanism more accurately.

Meanwhile, the RSCT can only obtain the macro-performance of asphalt mixtures but cannot elucidate the micro-properties of asphalt mixtures. An asphalt mixture is a heterogeneous material composed of aggregates, asphalt binders, and air voids. It is helpful in dealing with rutting disease, by investigating the micro interactions between components of asphalt mixtures subjected to loading. In studies that characterized micro-morphology, the discrete element method (DEM) has provided technical support and has been widely used recently [20,21,22]. The DEM model takes one particle as a unit and gives different properties to different particles. Therefore, the mechanical behavior and motion state of each asphalt mixture particle may be analyzed according to Newton’s second law [23,24]. Liu et al. developed a simulation of realistic particle shapes in mixtures through a MATLAB-based imaging process and PFC-based FISH codes [25]. Li et al. carried out an indirect tensile fatigue test of asphalt mixtures simulated with an image-based discrete element model [26]. Papagiannakis studied the plastic deformation behaviors of asphalt mixtures subjected to loading using DEM [27]. However, most existing studies consider the actual interface morphology of the mixture [28,29,30,31], and the corresponding virtual tests may only be used once. The relationship between the displacement of particles with different sizes and the macroscopic deformation has yet to be studied. Therefore, it is necessary to establish a new discrete element model for further analysis.

In order to clarify the rutting mechanism of asphalt mixture, this study reveals the rutting deformation mechanism of asphalt mixture from the micro-perspective, by developing a new rutting tester and combining it with the discrete element software. In addition, the movement characteristics of different particle size aggregate are expounded. The specific tasks of this study are as follows:

1. A more practical rutting tester is developed considering the shortcomings of existing testers.
2. A two-dimensional RSCT virtual test is built based on the discrete element method, and the validity of the virtual test is verified.
3. The microscopic response of the numerical model is analyzed to study the formation mechanism of rutting, and the corresponding guidance is obtained.

## 2. Materials

### 2.1. Asphalt

Basic asphalt, with a density of 1.03 g/cm^3^, is used in this study, with a penetration grade of 60/80, called “Pan Jin” basic asphalt. The technical parameters of the materials were tested according to the Chinese Test Methods of Bitumen and Bituminous Mixtures for Highway Engineering (JTG E20-2011). All parameters are shown in Table 1.

### 2.2. Aggregate

The aggregate and mineral powder used in this study is limestone, which was acquired from the Jiutai Stone Factory. The nominal maximum size of aggregates was 16 mm. The technical parameters of the materials were tested via experimentation according to the Chinese Test Methods of Aggregate for Highway Engineering (JTG E42-2005). All parameters are shown in Table 2, Table 3 and Table 4.

### 2.3. Preparation of Specimens

Two kinds of specimens were used: the asphalt mixture and asphalt mastic specimens. The maximum nominal size of the dense-graded asphalt mixture used in this paper was 16 mm, named AC16. This type of asphalt mixture is the most commonly used asphalt surface material in China. The specimens would then be used to test the rutting performance. The asphalt mastic specimens would be used to obtain the microscopic parameters used in the Burgers model.

#### 2.3.1. Preparation of Asphalt Mixture

**Figure 1:**
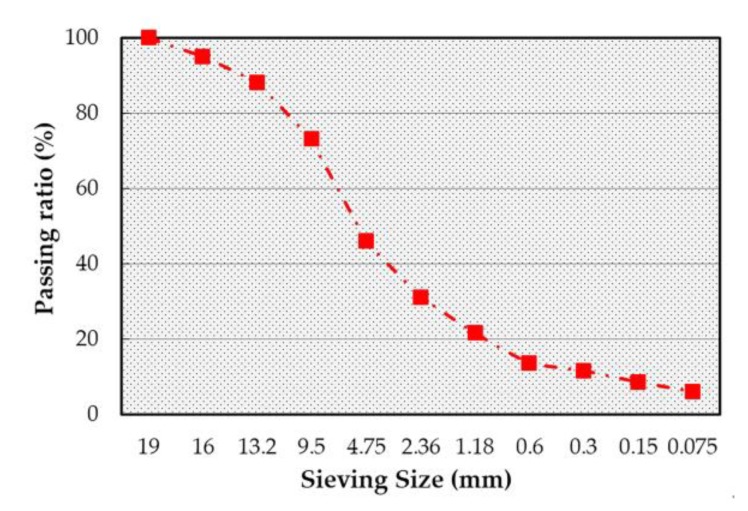
Gradation of AC16.

In the indoor tests, asphalt mixture specimens with a target porosity of 4% were prepared according to JTJ 052-2000. The optimized asphalt–aggregate ratio determined by the Marshall design method was 4.8%. Figure 1 shows the aggregate gradation and asphalt contents of AC16.

#### 2.3.2. Preparation of Asphalt Mastic

**Figure 2:**
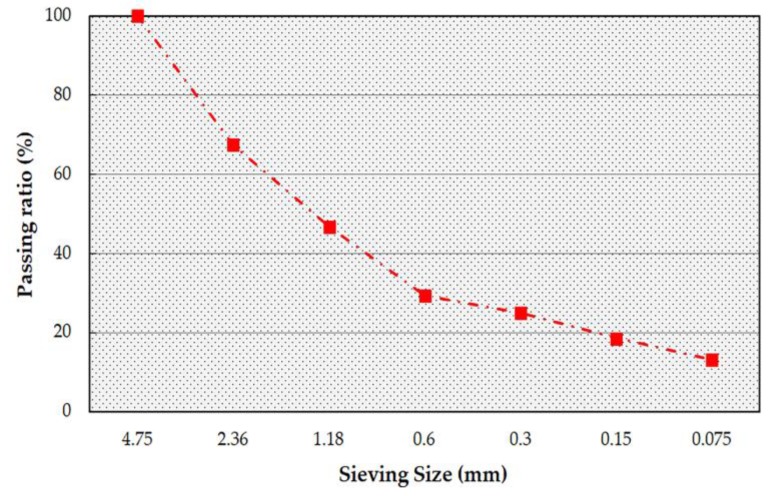
Gradation of asphalt mastic.

According to the aggregate gradation of asphalt mixtures, the aggregate gradation of asphalt mastic is shown in Figure 2. Marshall specimens of asphalt mastic were prepared according to the requirements of the dynamic creep tests to attain the macroscale parameters. The asphalt content of asphalt mastic was determined through the direct proportional relationship of the specific surface area between the asphalt binder and fine aggregates, which was 10%.

## 3. Methods

The rutting mechanism was studied using two laboratory rutting tests and a numerical simulation test. The two laboratory rutting tests used in this study were the WTT and the RSCT test, and the numerical simulation test was a discrete element rutting test based on the RSCT test. In the specifications of both America (AASHTO Guide for Design of Pavement Structures) [32] and China (JTG D50-2017), it has been proposed that permanent deformation of high-grade asphalt pavement over 15 mm affects the normal driving of vehicles. Therefore, the test ends when the rut deformation reaches 15 mm. Each test (the WTT and the RSCT test) was carried out three times. In order to ensure the reliability of the experimental data, the average value of the experimental data was used to express the results.

### 3.1. The Wheel Tracking Test 

The WTT was conducted and compared with the RSCT test to verify the effectiveness of the RSCT test. The WTT was carried out according to the Chinese standard GB/T0719. The experimental temperature applied in this test was 60 °C. The load pressure was 0.7 MPa, and the loading distance of the test wheel was 230 mm, with the linear reciprocating motions of the test wheel being 42 times per minute.

### 3.2. The Reduced Scale Circular Track Test

In all rutting tests, the full-scale test was deemed to be the most practical. However, this test is expensive and difficult to repeat. Therefore, laboratory tests were used to generally study the rutting performance of the asphalt mixture. The loading mode of the laboratory tests was usually linear reciprocating loading. The corresponding loading speed was different from the actual situation and was not constant, which would cause various deformations in the specimen at different positions. Considering the above, a new laboratory rutting tester called the RSCT tester was independently developed. The loading mode of the tester was annular loading to ensure that the deformation of the specimen was the same everywhere. The loading speed and size of the instrument may also be adjusted to meet the actual road conditions well.

#### 3.2.1. Compositions of the Tester

**Figure 3:**
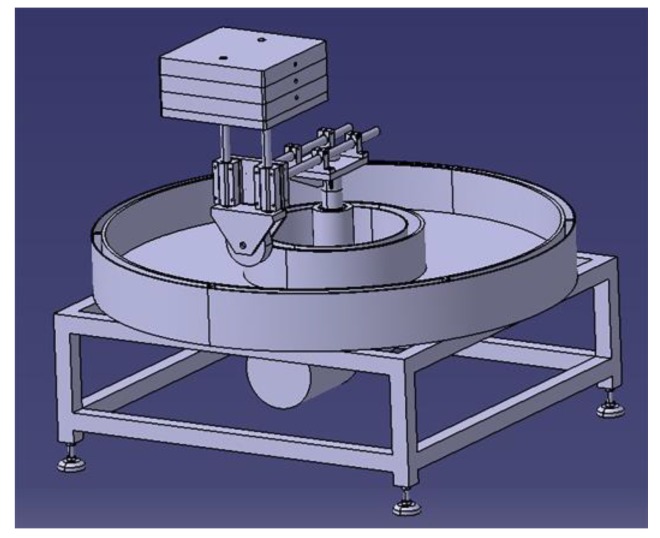
The RSCT tester.

The self-developed tester consists of four parts: the power system, the environmental simulation system, the loading system, and the monitoring system. The power system is used to provide loading power and control the loading frequency, and the environmental simulation system controls environmental conditions such as temperature and humidity. The loading system is used to control the loading position, weight, and size. The monitoring system is used to record the rutting depth and temperature. The corresponding tester is shown in Figure 3.

#### 3.2.2. Parameters of the Tester

The functions of the asphalt mixture preparation and rutting monitoring are integrated in the tester. Thus, the compaction times and load conditions need to be determined.

The asphalt mixture must first be compacted in the disc to form the specimen. The theoretical volume of the specimen was calculated according to the design height of the specimen, which was 5 mm. Therefore, the quality of the asphalt mixture may be obtained by density conversion. Thereafter, the mixed asphalt mixture was placed in the disc evenly and compacted by the test wheel with a width equal to the specimen. The compaction load was 65 kg, and following 50 times of compaction, four average positions of the specimen were selected to measure the compaction height. The height is shown in Table 5, which shows that the compaction heights reached the theoretical design height with an error of less than 4%. The specimen was taken out for porosity inspection, and the porosity was determined to be 5.1, satisfying the specification requirements. The above procedure indicated that this compaction was able to meet the requirements of the test and may be used to compact asphalt mixtures in the following test.

The load conditions consisted of load and temperature. The temperature was controlled at 60 °C, and the load was set to 70 kg, making the load pressure 0.7 MPa.

#### 3.2.3. Test Procedure

According to JTG E20-2011, the test procedure is as follows:

1. The resistance wire in the surrounding ring is heated to ensure a temperature of 120 °C.
2. The asphalt mixture, mixed according to JTG E20-2011, is evenly placed in the disc and tamped. Subsequently, the width of the test wheel is adjusted to the same width as the specimen and the asphalt mixture is compacted with a 65 kg load 50 times.
3. The test wheel is adjusted to a wheel with a width of 5 cm. The temperature is controlled at 60 °C by an environmental simulation system and held for 5 h. Finally, the test is carried out after the load is adjusted to 70 kg.

### 3.3. Numerical Simulation Test

Based on the RSCT test, a numerical simulation test was established. All aspects of the numerical simulation test are consistent with the RSCT test. The details are as follows: In terms of specimens, the gradation of specimen in the numerical simulation test is consistent with that in the RSCT test. The 2D specimen in the numerical simulation test is a full-size replica of the cross section of the RSCT test specimen. In terms of loading conditions, both the load and the load time are the same as those in the RSCT test. The temperature condition in the RSCT test can be achieved in the numerical simulation test by giving the micro-parameters at the corresponding temperature to a virtual specimen. A brief introduction is given below regarding the basic assumptions and contact models of the numerical simulation test.

#### 3.3.1. Basic Assumptions

In the discrete element analysis, particles were used to represent substances. According to the actual structure of the asphalt mixture, a discrete element model was then established. It was assumed that fine aggregate and mineral powder were wrapped in the asphalt, forming a homogeneous asphalt mastic material. Hence, the particles of the model may be divided into two parts: the coarse aggregate and asphalt mastic parts. 

In the process of building the discrete element model, it is very important to accurately simulate the actual situation of the asphalt mixture. Numerous studies have demonstrated that the shapes and distributions of coarse aggregate confer a great influence on the performance of the asphalt mixture [33,34]. The unreasonable shape and distribution of the aggregate will lead to the simulation deviation from the reality, resulting in a large error [35]. Therefore, the shapes and distributions must be considered for a high-precision rutting simulation. It is obvious that the original simple circle cannot represent the actual shape of the aggregate. Hence, the aggregates with a complex shape and uniform distribution are used to form the virtual asphalt mixture specimen [30]. The gradation of the virtual asphalt mixture is consistent with the RSCT test. Accordingly, the basic assumptions are as follows:

1. The shape of the coarse aggregate is assumed to be an irregular pentagon. In this paper, an irregular pentahedron’s aggregate clump is composed of several original round particles using FISH codes.
2. The distribution of the coarse aggregate is random.
3. Due to the fact that the coarse aggregate will not deform when subjected to loading, the coarse aggregate is assumed to be a homogeneous material with sufficient strength and stiffness.
4. Due to its recoverable capacity under high temperatures, the plastic deformation of asphalt mastic is very small, which can be ignored. Asphalt mastic has both elasticity and viscosity under high temperatures. It is unreasonable to treat asphalt mastic as a single elastic or viscous body. Therefore, asphalt mastic is assumed to be a homogeneous viscoelastic material. The Burgers model was utilized to describe the properties of asphalt mastic. The parameters would then be obtained using the dynamic creep test.

#### 3.3.2. Contact Models

Contacts and contact models in the discrete element model must be set up according to the assumptions above. In this study, three contact models exist:

1. The contact model of coarse aggregate particles is set up as a linear contact model.
2. The contact between the asphalt mastic particles and the coarse aggregate particles is set up as a linear contact bond model.
3. The contact model of asphalt mastic particles is set up as a Burgers model.

## 4. Results and Discussion

### 4.1. The Reduced Scale Circular Track Test 

**Figure 4:**
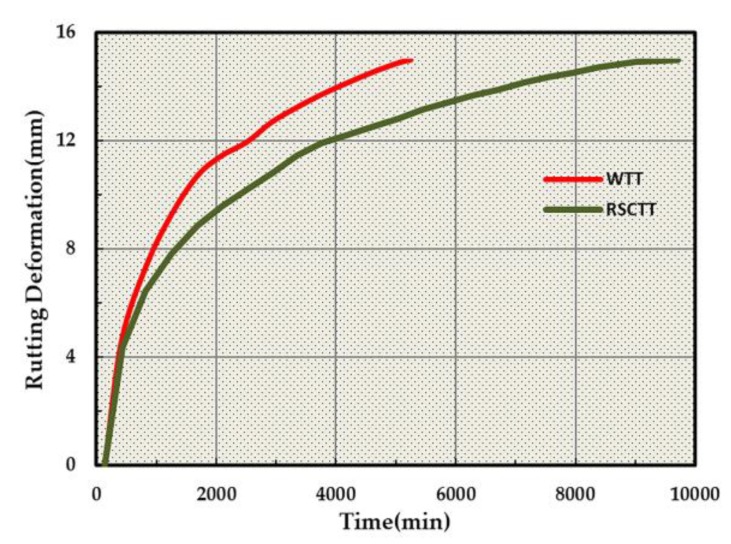
Deformation curve of the WTT and the RSCT test.

After the specimens were kept at 60 °C for 5 h, the tests were performed. The tests ended when the rutting deformation reached 15 mm. At this time, the load times measured by the HYCZ-5A tester (a rut tester for WTT) were 5219, and the load times measured by the RSCT tester were 9179. The rutting deformation curves of the two rut testers under the same load action times are shown in Figure 4.

In Figure 4, the formation rate of permanent deformation increases first and then slows down with the increase in loading times. The rut deformation of the RSCT test is always smaller than that of the WTT when the load and load times are the same. The load times of WTT account for 9.22% of the total times, and that of the RSCT test account for 5.83% when the deformation reached 5 mm. The load times of the WTT and the RSCT test account for 30.29% and 23.51%, respectively, when the deformation reached 10 mm. Both the ratio and growth ratio of the RSCT test is smaller than that of the WTT. Concerning the quantitative analysis of deformation, a logarithmic curve was used to fit the data. The fitting formula of the curve is shown in Table 6, and the determination coefficients of the two were determined to be 0.9785 and 0.9715, respectively, indicating that the degree of curve fitting is high.

The *a* of the RSCT test curve is 3.5594, which is smaller than that of the WTT curve of 4.136. The value of *a* represents the deformation rate. The smaller the value, the more load times needed to reach a certain deformation. Therefore, the rut deformation curve of the RSCT test is always below the WTT curve. A smaller *a* value indicates that the RSCT test specimen has a smaller deformation under the same load times.

There are generally two causes for differing performances in asphalt mixtures: internal and external causes. Internal causes are known as the performances of asphalt mixture materials. Both the materials and gradation used in the two rut testers were the same. The errors in the actual test operation were ignored. Therefore, the difference of the two testers in the results was not due to internal causes. The external causes are the test environment and load parameters. There are only two different external causes in the two rut testers: the speed and form of the load. The load linear speed of the traditional rut tester was 0.58 km/h, while that of the new rut tester was 3.69 km/h. The load linear speed of the new tester was observed to be closer to the actual vehicle speed. The practical experience shows that the lower load speed will produce a larger permanent deformation. Therefore, the specimen in the new rut tester possessed a smaller deformation under the same load times. The second external cause is the form of the load. The specimen surface of the traditional rut tester was squared, and the loading wheel performed a reciprocating linear motion during the test. This form of the load will lead to the accumulation of asphalt mixture at the front and rear edge of the specimen as well as different deformations at each position of the specimen. The load form of the new rut tester is annular load. Therefore, there will be no accumulation in mixtures, and the rut deformation of the whole specimen will be basically the same. 

In view of the above two points, the load of the new rut tester more closely conforms to that of the actual pavement. Hence, the new tester may more reliably evaluate the rutting potential of asphalt mixtures.

### 4.2. The Virtual Rutting Test 

#### 4.2.1. Preparation

During the numerical simulation, the virtual test was established using the discrete element software PFC2D. The asphalt mastic was considered to be a homogeneous viscoelastic material. The Burgers model parameters for the contacts between the asphalt mastic elements were obtained via the dynamic creep test.

**Figure 5:**
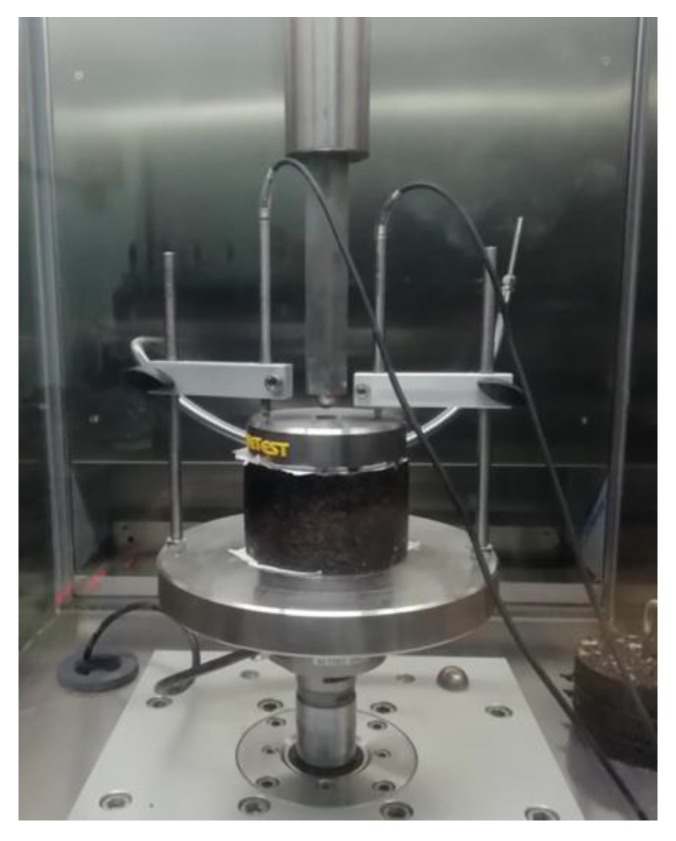
Dynamic creep test.

The instruments used in the test were the DTS-30. The Marshall specimen was compacted on both sides 75 times. The cylinder specimen, with a diameter of 101.6 mm and a height of 63.5 mm, was prepared for the uniaxial creep tests [36,37,38], as shown in Figure 5.

The experimental temperature applied on the creep test was 60 °C, consistent with the experimental temperature applied in the rutting test. The specimen was thermally insulated for 5 h. A square wave pressure with a frequency of 0.5 Hz and a load of 100 kPa was applied on the specimen. Every 2 s was considered a cycle, and there were 1800 cycles. In order to eliminate errors caused by the device, a preload of 10 kPa was first applied on the specimen for 5 min.

**Figure 6:**
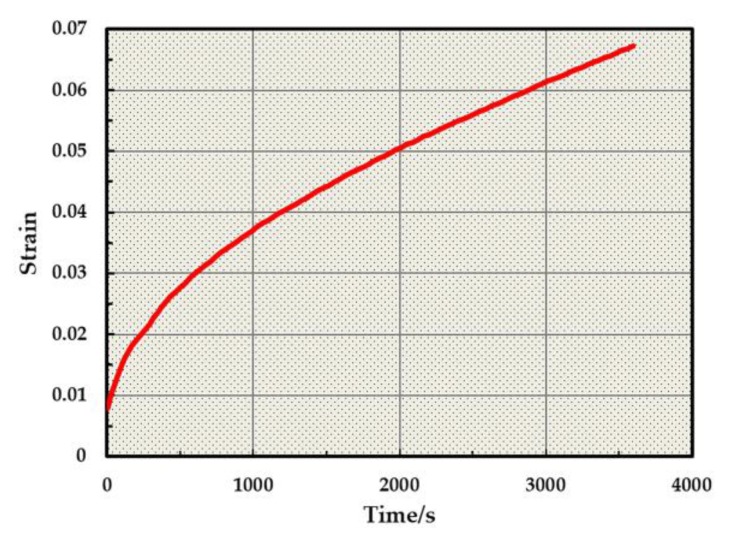
Creep curve of AC-16 asphalt mastic at 60 °C.

The fitting curve of the dynamic creep test was then recorded, as shown in Figure 6. The Burgers model is a four element viscoelastic model, composed of a Maxwell model and a Kelvin model in series. Its creep equation can accurately describe the stress–strain relationship of the asphalt mixture. The parameters of the Burgers model were obtained by fitting the creep equation (Equation (1)) of the Burgers model and are shown in Table 7. The value of the correlation coefficient R was determined to be 0.999444, and the value of the determination coefficient R^2^ was 0.998888. The fitting effect was observed to be ideal.

(1) $$\varepsilon \left(t\right)=\frac{\sigma }{E_{1}}+\frac{\sigma }{E_{2}}+\frac{\sigma t}{\eta_{1}}-\frac{\sigma }{\eta_{2}}e^{-E_{2}t^{\prime }/\eta_{2}}$$

where *ε* is the strain, *σ* is the stress, *t’* is the loading time, *E_1_* is the Maxwell elasticity coefficient, *η_1_* is the Maxwell viscosity coefficient, *E_2_* is the Kelvin elasticity coefficient, and *η_2_* is the Kelvin viscosity coefficient.

#### 4.2.2. Parameters and establishment of the model

● The load time parameters

The per unit length load time of the new rutting tester for 1 h is calculated as follows:

(2) $$T=\frac{3600\cdot l}{s}=32.757s$$

where *l* is the contact length of the area between the test wheel and the specimen, and *s* is the one-time loading distance of the test wheel.

According to the previous RSCT test, the load time was found to be 328 min when the deformation was 15 mm. Therefore, the total load time of the virtual rutting test was determined to be 0.0179 s, based on the time–temperature equivalence principle with a conversion coefficient of 10^4^.

● The macro contact parameters between the coarse aggregate particles. 

Aggregates were considered as elastic materials without deformation. The desired modulus of the coarse aggregates was 55.5 GPa [39]. The damping force was ignored as the contact force between aggregate particles possessed no viscosity.

● The macro contact parameters between the asphalt mastic particles.

Based on the time–temperature equivalence principle and the transformation equations [40], the micro-parameters of the asphalt mastic particles were calculated using the following formula, as shown in Table 8.

(3) $$\{\begin{array}{l} K_{mn}=E_{1}t,C_{mn}=\eta_{1}t,K_{kn}=E_{2}t,C_{kn}=\eta_{2}t\\ C_{ms}=\frac{C_{mn}}{2\left(1+\nu \right)}=\frac{\eta_{1}t}{2\left(1+\nu \right)},K_{ms}=\frac{K_{mn}}{2\left(1+\nu \right)}=\frac{E_{1}t}{2\left(1+\nu \right)}\\ C_{ks}=\frac{C_{kn}}{2\left(1+\nu \right)}=\frac{\eta_{2}t}{2\left(1+\nu \right)},K_{ms}=\frac{K_{kn}}{2\left(1+\nu \right)}=\frac{E_{2}t}{2\left(1+\nu \right)} \end{array}$$

where *t* is the disk thickness.

● The macro contact parameters between the coarse aggregate particles and asphalt mastic particles.

Because bond failure will not occur in an actual situation, the tensile strength and shear strength in this linear contact bond model were set to a larger value [25]. The calculation results of the normal stiffness and tangential stiffness are shown in Table 9.

● Establishment of the rutting emulation test.

**Figure 7:**
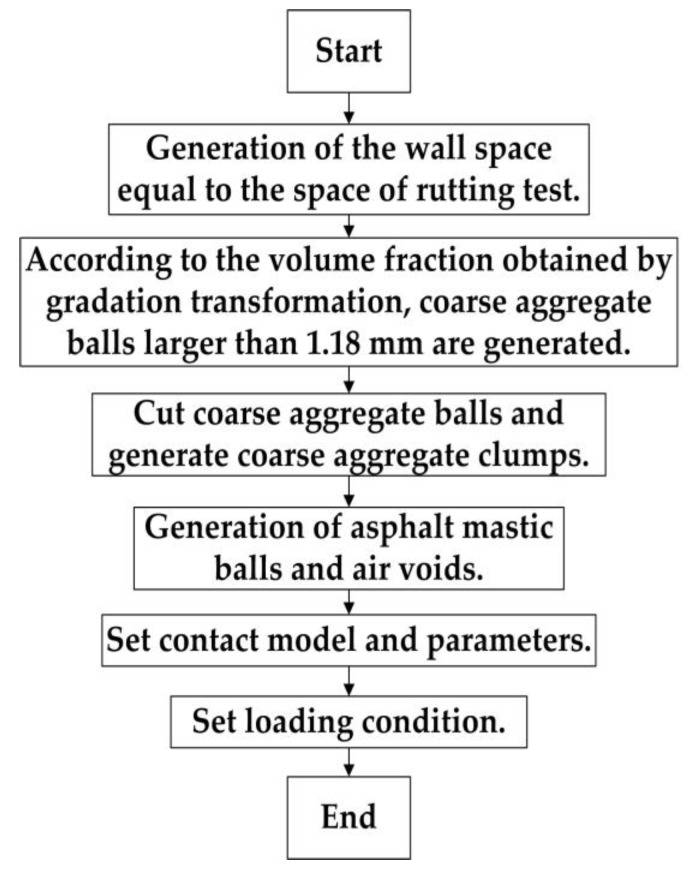
Establishing steps of the model.

The specific steps in establishing the model are shown in Figure 7.

In view of the stability of mechanical properties of materials, as well as the computational efficiency of the PFC software, the asphalt concrete in PFC was composed of mesoscale phases including coarse aggregates and asphalt mastics. More specifically, the asphalt mastics were regarded as homogeneous particles composed of an asphalt binder, mineral filler, and fine aggregate, with a nominal size smaller than 2.36 mm.

First, a rectangular rutting test area of 300 × 50 mm was established, where coarse aggregate balls in different sizes and random locations were generated according to the preceding gradation. FISH codes were then used to cut the circular coarse aggregate balls into an irregular pentahedron, in order to simulate the actual shape of the coarse aggregate. After recording information pertaining to the position and shape of the balls larger than 2.36 mm, all balls were deleted, and balls with a radius of 1 mm were generated to fill the entire test area. The balls located at the position of the coarse aggregate, after being cut, formed clumps without deformation. The remaining balls represented the asphalt mastic. A certain number of asphalt mastic balls were then removed as air voids, and the contact model and parameters were set.

The loading condition of the rutting test was 0.7 MPa. To simulate the test, the loading condition of the discrete element model was also 0.7 MPa. Specifically, 250 balls with a radius of 1 mm were used to construct the loading clump tool (25 × 10) of a model possessing an adequate level of strength and no deformations. After setting the constant loading pressure, in order to avoid the displacement of the loading clump tool in the loading process, caused by the uneven distribution of asphalt mixture aggregate, the x-axis of the loading clump tool was fixed to ensure the accuracy of the loading position.

**Figure 8:**
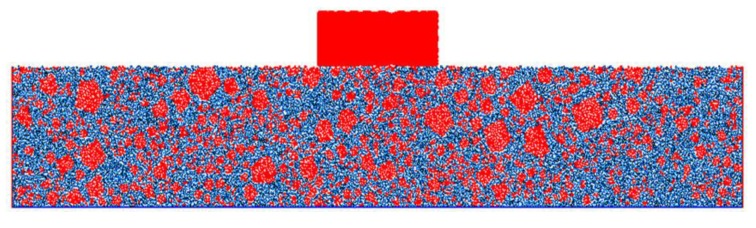
The DEM of the virtual track test.

At this time, the virtual rutting test established according to the given procedure is shown in Figure 8. The model is mainly composed of coarse aggregate clumps (red pentagons), asphalt mastic balls (blue balls), and the loading clump (red rectangle). The number of coarse aggregate clumps is shown in Table 10.

The real test loading time will be transformed by the time–temperature equivalence principle, as the loading time of the discrete element model should not be too long. The Burgers model’s parameters were also reduced so as to improve the efficiency of the model. The time–temperature conversion coefficient was set to 10,000 in order to maintain the model’s accuracy, as demonstrated by numerous previous studies.

#### 4.2.3. Model Validation

**Figure 9:**
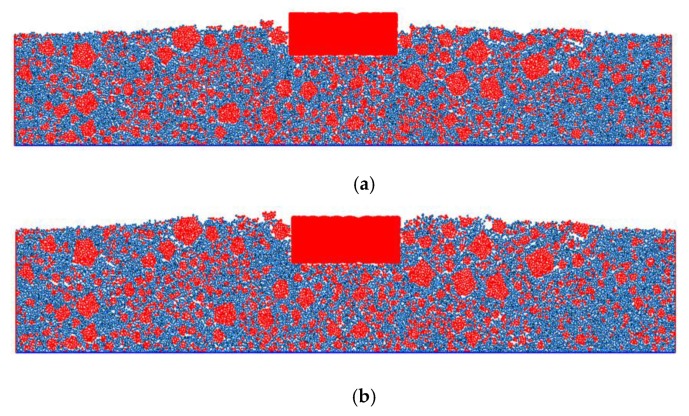

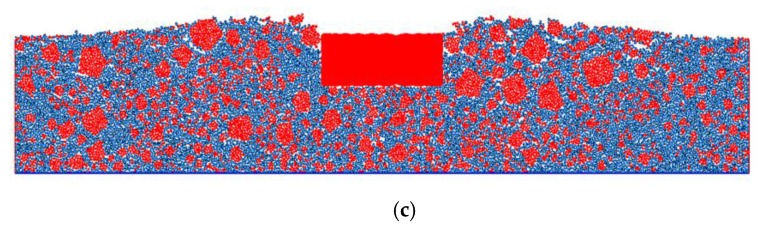
(**a**) Rutting deformation of the virtual track test at 110 min. (**b**) Rutting deformation of the virtual track test at 220 min. (**c**) Rutting deformation of the virtual track test at 328 min.

The HISTORY module in PFC2D was used to monitor the change in rutting deformation. The rutting change of the specimen was recorded and is shown in Figure 9.

**Figure 10:**
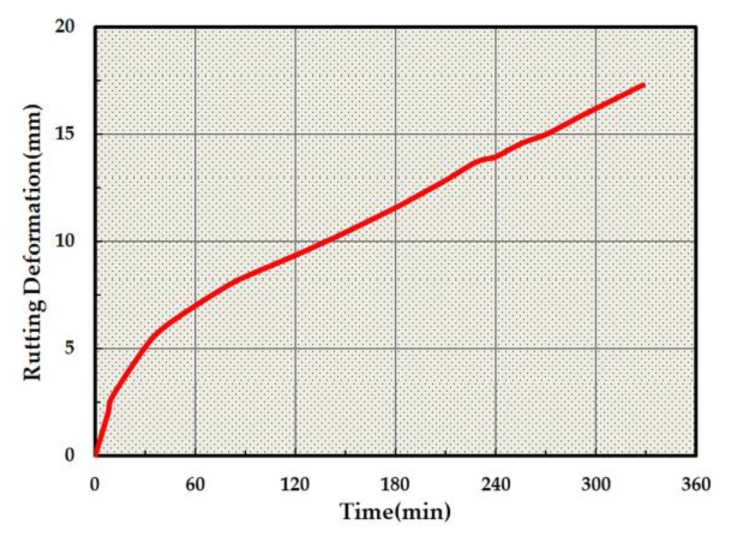
Deformation curve of the virtual track test.

According to Figure 9, the deformation is seen to grow with the increase in loading time. From a micro perspective, the rutting deformation was observed to be the slip of asphalt mixture particles. From the macroscopic point of view, the deformation of the loading area is obviously larger than that of the unloaded area. Combined with Saint Venant's principle, it can be seen that the stress in the loading area is large and easily causes deformation, while the stress far away from the loading position is small and no obvious deformation occurs. In addition, the asphalt mixture humps on the left and right sides of the loading position are obvious in the figure. The hump in the opposite direction of the load is due to the fact that the asphalt mixture at the loading position is squeezed by load and flows far away from the load position. The hump is consistent with the actual shape of pavement rutting. In order to further quantify this degree of coincidence, the deformation curve recorded by the HISTORY module is shown in Figure 10, and the results of the virtual test are compared with that of the RSCT test.

The final deformation of the virtual track test was found to be 17.31 mm, with an error of only 15.4% when compared to that of RSCT. Figure 10 depicts the increase of the loading time, where the deformation is observed to be growing, but the growth rate gradually becomes slower. This evaluation aligns with the actual change in rut deformation.

**Figure 11:**
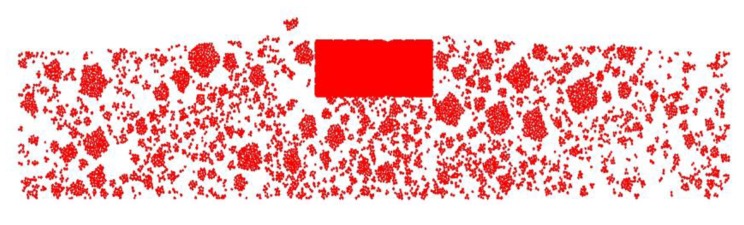
Coarse aggregate distribution after loading.

Figure 11 shows the location of the coarse aggregate particles after loading. Red represents the aggregate particles. It is clearly evident that the density of aggregate particles under the loading position is larger than that of the other position. The density of the aggregate particles gradually decreased from the loading position to the edges of the specimen. The density of the mastic particles in the raised position is larger than that of the other position. This phenomenon is consistent with the rutting sample extracted from the actual pavement, proving the validity of the model [41,42].

According to the aforementioned data, the validity of the model was verified to be adequate.

#### 4.2.4. Analysis and Discussion

**Figure 12:**
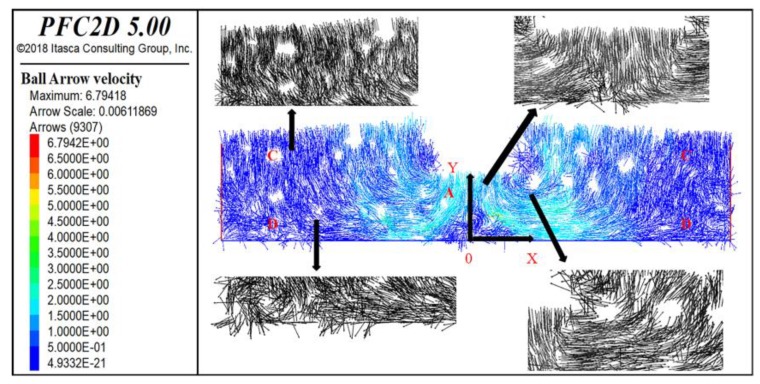
Displacement vector of particles.

**Figure 13:**
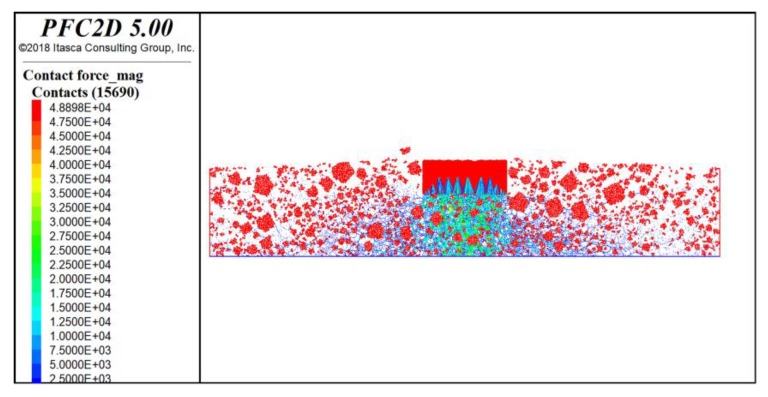
Contact force of particles.

During the loading process, it was found that, with the increase in loading time, the aggregates exhibited obvious sliding behaviors: the aggregates on both sides of the loading wheel edge would turn outwards and move upwards gradually, and the aggregate under the loading wheel would move downward. Asphalt mastic particles moved in eddy currents by the driving of aggregate particles. In order to clearly analyze this event, the displacement vector and contacts of the particles in the coordinate system were plotted, as shown in Figure 12 and Figure 13. The coordinate system was a plane rectangular coordinate system set according to the following: the coordinate zero point was chosen as the center position of the bottom of the specimen; the positive direction of the y-axis is upward; and the positive direction of the x-axis is towards the right.

In Figure 12, the direction of the arrow indicates the direction of the particles’ displacement, and the color indicates the value of the displacement vector. Here, red indicates the maximum displacement vector, and blue signifies the minimum displacement vector. It can be seen from Figure 12 that four main kinds of particle displacement exist in the A, B, C, and D regions of the specimen, respectively. Two kinds of displacements appeared near the loading position, with the first having occurred under the loading wheel (A). Due to the downward action of the loading wheel, this kind of displacement is mainly observed to be negative to the y-axis. The displacement vector was next to the second kind of particle displacement, which occurred on both sides of the loading wheel edge (B). This kind of displacement vector is an eddy current and is obviously larger than the other displacements. This is because the particles have both vertical displacement as well as lateral displacement. The other two kinds of displacements occurred in the specimen far away from the loading wheel. These displacements were small and decreased with the distance of particles away from the wheel. In the upper part of the specimen away from the loading wheel (C), the particle displacement mainly accounted for a small amount toward the positive direction of the y-axis. In the lower part of the specimen far away from the loading wheel (D), the particle displacement was also small and transverse, and very distant from the loading wheel. The four kinds of displacement vectors show the formation of the ruts and explain the uplift of the wheel track edge during the rutting process perfectly [41,42]. In Figure 13, the value change of contact force is consistent with that of the displacement vector at the corresponding location, which explains the state of the particle displacement vector in the four regions in Figure 12: the macro load that a particle bears is the contact force with its surrounding particles in the microstructure. The larger the contact force is, the larger the load it bears, and the larger the displacement vector is. For example, in Figure 13, it can be seen that the contact force of particles in area A is the largest. Therefore, it can be seen that the displacement vector of particles in area A is also the largest in Figure 12. The same is true for particles in other regions.

According to the above observations, the displacement trend of the particles was briefly analyzed to study the mechanism of rutting formation. If the material properties, construction technology, and natural environments are the same, the gradation will have a greater impact on the rutting performance of the asphalt mixture. The influence of the particle size on rutting deformation was then quantitatively analyzed. The displacement of the aggregate particles after loading is represented by FISH codes, and the contribution rate (CR) of the coarse aggregates for rutting deformation is calculated as follows:

(4) $$\bar{D_{m}}=\frac{\sum_{i=1}^{n}D_{i}}{n}$$

(5) $${\mathrm{CR}}_{m}=\frac{\bar{D_{m}}}{\sum_{i=1}^{m}\bar{D_{m}}}\times 100\%$$

where $\bar{D_{m}}$ is the average absolute displacement of aggregates in m-grade, and *n* is the number of aggregates in m-grade.

**Figure 14:**
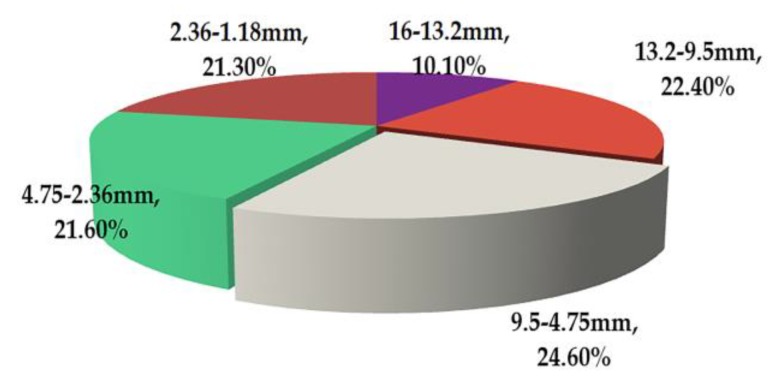
Contribution rate of coarse aggregates with different sizes for rutting deformation.

Figure 14 shows the CR of coarse aggregates of different sizes for rutting deformation. The CR of particles with diameters larger than 4.75 mm was 62.9%, illustrating that the primary cause of rutting deformation was due to the displacement of aggregates of large sizes. The coarse aggregate in asphalt mixture plays an important role in bearing load [43,44]. The skeleton structure of the asphalt mixture is mainly composed of large size aggregates (aggregates larger than 4.75 mm). It can be seen from Figure 14 that the CR values of these large size aggregates are large. The CR of aggregate particles with diameters ranging from 9.5 to 4.75 mm was found to be 24.6%, which is 1.1–2.4 times that of the other aggregates. This is because the aggregates with diameters ranging from 9.5 to 4.75 mm are the main constituent aggregate of skeleton structure (the number of 9.5–4.75 mm aggregates is 42, accounting for 80.77% of the total number of large size aggregates). Moreover, the CR of aggregate particles with diameters between 16 and 13.2 mm was 10.1%, signifying the smallest amount, where the CR of the other aggregate particles was also almost the same. This was because all particles of 16–13.2 mm were in the part of the specimen with a small particle displacement (area C in Figure 12). The above data shows that the average absolute displacement of aggregate particles with diameters from 9.5 to 4.75 mm is 1.1–2.4 times that of other aggregate particles. Therefore, it can be preliminarily inferred that aggregates with diameters from 9.5 to 4.75 mm have the largest contribution to pavement rutting. This point has been confirmed in the design of asphalt mixture gradation by the Bailey method. One of the key indexes of the Bailey method is the CA (coarse aggregate) ratio, which is mainly related to the passing amount of NMPS/2 (nominal maximum particle size/2) and PCS (primary control sieve) [45,46]. In this paper, NMPS is 16 mm. NMPS/2 is 8 mm, which is close to 9.75 mm. PCS is 3.52 mm, which is close to 4.75 mm. These two key sieve sizes of the Bailey method are consistent with the particle size with the largest CR value in the previous conclusion. The effectiveness of the CR index is verified, and the amount of 9.5–4.75 mm aggregate deserves special attention [47,48,49].

**Figure 15:**
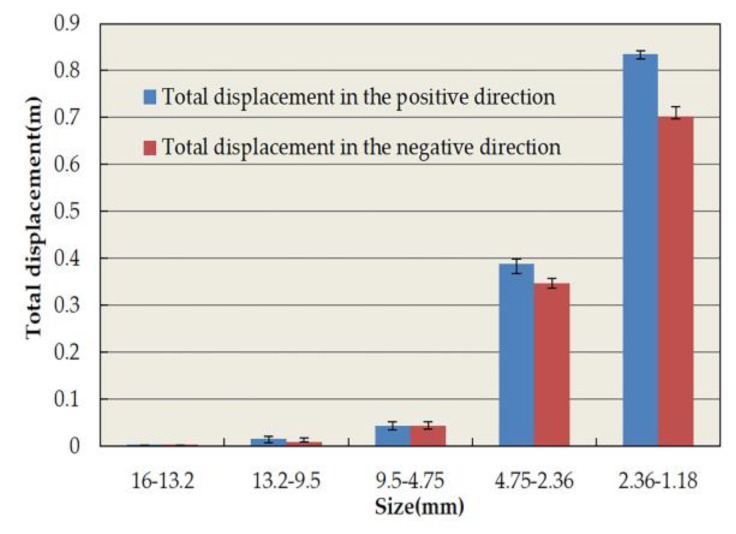
Total x-axis displacement of coarse aggregates.

**Figure 16:**
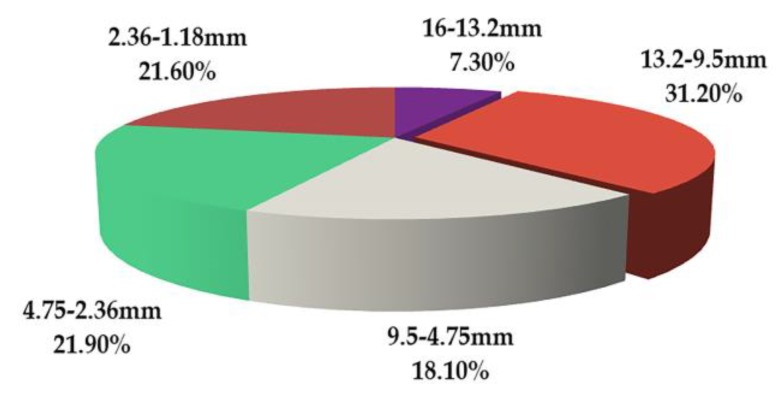
Contribution rate of coarse aggregates of different sizes for x-axis rutting deformation.

Figure 15 and Figure 16 depict the statistical displacement data of aggregate particles on the x-axis displacement. In Figure 15, it is apparent that all particles possess x-axial displacement. Essentially, all particles tend to move towards the edge of the specimen, behaving in accordance with the actual spatial movement of asphalt mixture particles. The total lateral displacement of the particles increases with the decrease in particle size. The number of aggregates increases rapidly with the decrease in aggregate size, which is shown in Table 10. Therefore, the total displacement of small aggregates is large. The displacement of aggregates in the positive and negative directions of the x-axis was approximately the same, except for particles with diameters ranging from 16 to 9.5 mm. This demonstrates the uniformity and randomness of aggregate distribution in the model. The reason for the difference is due to the small number of particles as well as the asymmetry of its distribution. According to Figure 16, the CR of particles with diameters from 13.2 to 9.5 mm is observed to be 31.2%, which was the largest measured CR. Moreover, the CR was 1.4–4.2 times that of the other aggregates, which may preliminarily imply that aggregates between 13.2 and 9.5 mm confer a greater influence in lateral rutting deformation. Because the specimen has a small transverse deformation, the x-axial CR value of 9.5–4.75 mm aggregates is small when these aggregates are the major part of the skeleton structure. This also shows that coarse aggregates play an important role in resisting permanent deformation [50,51]. In addition, compared with the CR value of aggregates with diameters larger than 4.75 mm for rutting deformation, the x-axial CR value of these aggregates only has a little change. A mere change of 0.6% indicates that the structure of the asphalt mixture remains stable in the transverse direction. This is caused by the fixed transverse boundary of the specimen, and the relevant behavior of the full-scale specimen needs further study.

**Figure 17:**
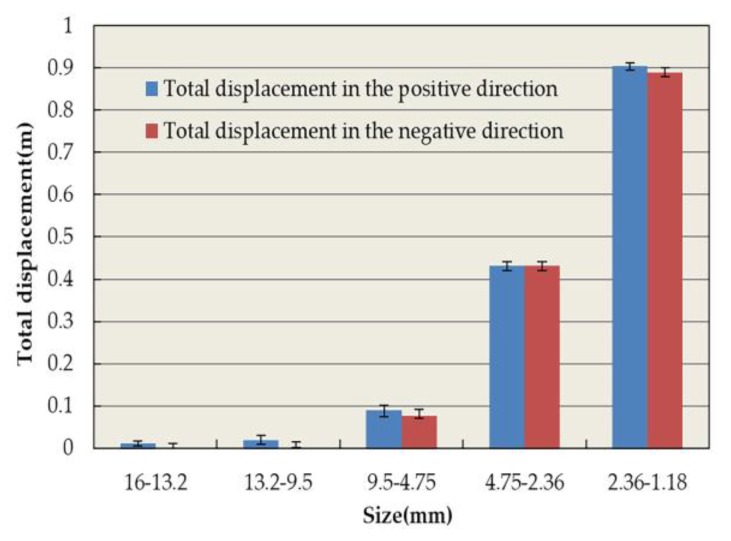
Total y-axis displacement of coarse aggregates.

**Figure 18:**
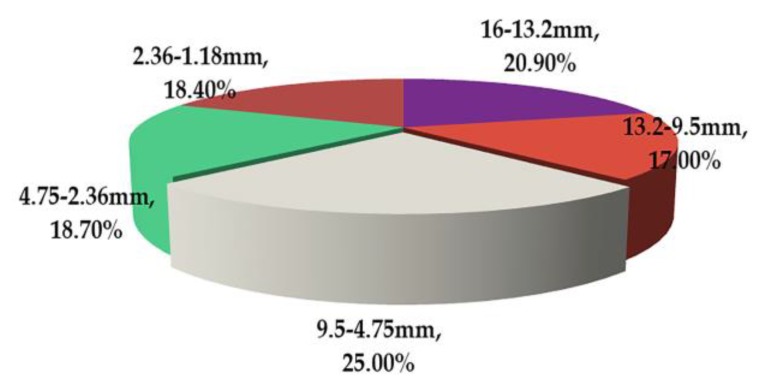
Contribution rate of coarse aggregates with different sizes for y-axis rutting deformation.

Figure 17 and Figure 18 illustrate the statistical displacement data of aggregate particles on the y-axis. Comparing the total longitudinal displacement of particles with different diameters, the smaller the particle size, the larger the displacement was found to be. This change is consistent with that of the lateral absolute displacement, as seen in Figure 15. The total longitudinal displacement of all particles in the positive direction is marginally greater than that in the negative direction, leading to the uplift morphology of rutting. This is also shown in Figure 18: compared with the CR value of aggregates with diameters larger than 4.75 mm for rutting deformation, the y-axial CR ratio of these aggregates has changed by 5.8%. This indicates that the structure of the asphalt mixture is unstable in the longitudinal direction, which leads to permanent deformation. In terms of the CR of coarse aggregates of different diameters in longitudinal rutting deformation, the CR of aggregates with diameters between 9.5 and 4.75 mm was observed to be the largest at 25%. The CR, accordingly, was 1.2–1.5 times that of the other aggregates, indicating that aggregates between 9.5 and 4.75 mm play an important role in longitudinal rutting deformation.

## 5. Conclusions

This paper presents a newly self-developed pavement rutting device as well as a discrete element model to analyze the mechanism of rutting. Based on completed analyses, the following conclusions may be drawn:

- Compared with the existing laboratory experiments, the RSCT test has a uniform deformation and a low deformation rate, showing a more practical and accurate test method for testing rut performance. The RSCT test can thus be widely used.
- A virtual numerical simulation test is established according to the RSCT test. The validity of the virtual rutting test using an irregular pentagon as the aggregate shape is verified. The micromechanical responses show that all mixtures within the stress range possessed a tension for displacement. The contact force and displacement of particles in the loading area were the largest and gradually diffused to the surrounding area.
- The asphalt mastic extruded by the displacement of aggregates demonstrated its rheological behavior, mainly resulting in the formation of rutting. The CR index shows that 4.75–9.5 mm aggregates make the largest contribution to rutting deformation. Special attention should be given to these aggregate amounts in the design of mixture grading.

In the future, further research can be carried out in three aspects. The aggregate in the virtual rutting test needs to be more consistent with the actual aggregate morphology. The rutting mechanism of asphalt mixture with different gradations need to be studied. A new mixture design method and a rutting prediction method may be proposed, based on the CR value.

## Figures and Tables

**Table 1:** Technical parameters of the asphalt.

Technical Parameter	Test Result	Specification Requirements	Test Procedure
25 °C penetration (0.1 mm)	61.9	60–80	T 0604-2011
Softening point (°C)	48.7	≥45	T 0606-2011
15°C Ductility (cm)	>150	≥100	T 0605-2011
Wax content (%)	1.9	≤2.2	T 0615-2011
Flash point (°C)	340	-	T 0611-2011
Solubility (%)	99.9	≥99.5	T 0607-2011

**Table 2:** Technical parameters of coarse aggregate.

Technical Parameter	Test Result	Specification Requirements	Test Procedure
Crushing value (%)	21.2	≤26	T 0316-2005
Los Angeles abrasion value (%)	25	≤28	T 0317-2005
Apparent specific gravity (t/m^3^)	2.79	≥2.6	T 0304-2005
Elongated particles content (%)	9.3	≤15	T 0312-2005
Water absorption (%)	0.61	≤2.0	T 0307-2005

**Table 3:** Technical parameters of fine aggregate.

Technical Parameter	Test Result	Specification Requirements	Test Procedure
Apparent specific gravity (t/m^3^)	2.71	≤2.5	T 0328-2005
Surface dry specific gravity (t/m^3^)	2.6	-	T 0330-2005
Specific gravity of gross volume (t/m^3^)	2.64	-	T 0328-2005
Mud content (%)	1.02	≤3	T 0333-2005

**Table 4:** Technical parameters of mineral powder.

Technical Parameter		Test Result	Specification Requirements	Test Procedure
Apparent specific gravity (t/m^3^)		2.83	≥2.5	T 0316-2005
Water absorption (%)		0.2	≤1	T 0316-2005
Appearance characteristic		No agglomeration	No agglomeration	T 0316-2005
Granular composition (%)	＜0.6 mm	100	100	T 0316-2005
	＜0.15 mm	98	90–100	
	＜0.075 mm	92	75–100	

**Table 5:** Compaction height of the RSCT tester.

Position	A	B	C	D
Compaction height (mm)	5.16	5.09	4.92	5.07

**Table 6:** The fitting formula of the curve.

Test	Logarithmic Equation y = *a*ln(x) − *b*	Determination Coefficient R^2^
RSCT test	y = 3.5594ln(x) − 17.478	0.9785
WTT	y = 4.136ln(x) − 20.352	0.9715

**Table 7:** Macro parameters of AC-16 asphalt mastic.

Parameter	E_1_ (MPa)	E_2_ (MPa)	η_1_ (MPa)	η_2_ (MPa)
Value	4.6581	6.5419	10.9929	2.1164

**Table 8:** Macro parameters of the Burgers model.

Parameter	K_kn_ (Pa·m)	C_kn_ (Pa·m·s)	K_mn_ (Pa·m)	C_mn_ (Pa·m·s)	K_ks_ (Pa·m)	C_ks_ (Pa·m·s)	K_ms_ (Pa·m)	C_ms_ (Pa·m·s)
Value (E6)	6.5419	0.2116	4.6581	0.1099	2.1806	0.0705	1.5527	0.3664

**Table 9:** Macro parameters of linear contact bond model.

Parameter	Kn (10^6^ N/m)	Ks (10^6^ N/m)
Value	10.1735	3.4437

**Table 10:** Number of coarse aggregate clumps.

Size (mm)	16–13.2	13.2–9.5	9.5–4.75	4.75–2.36	2.36–1.18
Number	3	7	42	293	620
